# Agreement Between Self‐Reported COVID‐19 and Dried Blood Spot Serology: A Cross‐Sectional Study

**DOI:** 10.1002/hsr2.72267

**Published:** 2026-04-08

**Authors:** Nicola Sheppard, Matthew T. C. Carroll, Brigitte M. Borg, Zheng Quan Toh, Paul V. Licciardi, Catherine L. Smith, Jillian F. Ikin, Michael J. Abramson, Karen Walker‐Bone, Tyler J. Lane

**Affiliations:** ^1^ School of Public Health and Preventive Medicine Monash University Melbourne Victoria Australia; ^2^ Monash Rural Health Churchill Monash University Churchill Victoria Australia; ^3^ Respiratory Medicine Alfred Health Melbourne Victoria Australia; ^4^ Infection, Immunity and Global Health Theme Murdoch Children's Research Institute Parkville Victoria Australia; ^5^ Department of Paediatrics University of Melbourne Parkville Victoria Australia

## Abstract

**Background and Aims:**

Identifying COVID‐19 cases with high accuracy is essential for epidemiological research on the pandemic's health effects. We aimed to investigate the agreement between a validated self‐report questionnaire for COVID‐19 and dried blood spot serology for SARS‐CoV‐2 antibodies.

**Methods:**

We conducted a cross‐sectional analysis of combined survey and SARS‐CoV‐2 antibody data. Between June and October 2023, 311 adults completed a validated self‐report COVID‐19 questionnaire and provided fingertip blood samples, which underwent Enzyme Linked Immunosorbent Assay to quantify IgG antibodies to SARS‐CoV‐2 nucleocapsid (N)‐proteins. We applied several statistical approaches to assess agreement: Cohen's κ of inter‐rater reliability; positive (PPV) and negative (NPV) predictive values; and logistic and linear regressions of the year of most recent self‐reported COVID‐19 on serostatus and N‐protein antibody concentrations.

**Results:**

Two‐thirds (203, 65%) of participants self‐reported a history of COVID‐19 whereas one‐third (98, 32%) were seropositive for SARS‐CoV‐2 N‐protein antibodies, reflecting only “fair” agreement (κ = 0.23 [95% CI 0.15–0.31]). Self‐reported COVID‐19 had a PPV of 41% and an NPV of 87% for SARS‐CoV‐2 seropositivity. PPV was low for those whose most recent self‐reported cases were in 2020–21 (36%) and 2022 (33%), but higher for 2023 (75%). Compared to participants with no self‐reported history of COVID‐19, those reporting SARS‐CoV‐2 infection in 2023 had 23 times greater odds of being seropositive (95% CI: 9.18–62.5) and had 1078% (617–1836%) higher N‐protein concentrations, after adjustment for confounders.

**Conclusion:**

While overall agreement self‐reported COVID‐19 and serostatus is only fair, there was a strong relationship exhibited between the two for recent self‐reported cases, when serology is most accurate. This suggests that self‐reported COVID‐19 is reasonably accurate for identifying people who have previously had COVID‐19 as well as determining roughly when infections occurred.

## 1 Introduction

In the 5 years since its emergence, COVID‐19 has attracted an unprecedented volume of research [1]. The urgency of sharing knowledge about COVID‐19, particularly during the early days of the pandemic, led to research being published faster than ever before [2], igniting concerns about its quality [1, 3, 4]. One of the most important public health metrics pertaining to COVID‐19 has been case numbers. Such data are vital to better understand SARS‐CoV‐2 infectivity and inform public health policy. Evaluating methods of COVID‐19 case detection remains important to understanding the reliability of data and to finding cost‐effective ways of monitoring the ongoing impact of COVID‐19.

Polymerase Chain Reaction (PCR) tests, which detect viral RNA in swabs of fluid taken from the nose and mouth during acute infection, are widely accepted to be the gold standard for COVID‐19 diagnosis [4, 5]. However, PCR sensitivity wanes over a few weeks [6], thus missing those who cannot access testing during acute infection. Furthermore, PCR testing programs that target symptomatic individuals miss between 32% and 65% of cases who do not exhibit symptoms [7, 8, 9]. Rapid Antigen Tests (RATs), which use a similar sampling method to PCR but detect viral proteins instead of RNA, can also be used at the time of acute infection. However, these have similar shortcomings to PCR testing and poorer sensitivity [10].

Serology testing, which identifies viral antibodies in a blood sample, can retrospectively identify SARS‐CoV‐2 infections. In contrast to PCR sensitivity, which wanes in the weeks following infection, the diagnostic accuracy of serology testing increases over the same period [6]. However, detectable COVID‐19 antibody levels in the blood also eventually wane over the months following infection, particularly in those with milder disease [11, 12, 13, 14].

Self‐report questionnaires are another means to retrospectively capture COVID‐19 infection. However, these too can yield a high rate of false positives as well as false negatives due to asymptomatic cases that are not tested [7, 8, 9, 15]. Alternatively, questionnaires that ask individuals to self‐report prior COVID‐19 diagnoses by PCR or medical opinion proved to be a valuable resource during the pandemic [16].

Our study evaluated the agreement between two methods of retrospectively detecting COVID‐19: a serology test measuring SARS‐CoV‐2 antibodies in dried blood spot (DBS) samples [17] and a validated self‐report questionnaire for COVID‐19 [18].

## 2 Methods

### 2.1 Sample

Participants were recruited from the Hazelwood Health Study Adult Cohort, which was established to investigate the long‐term health effects of the 2014 Hazelwood open‐cut coal mine fire in the Latrobe Valley, southeastern Australia [19]. Eligible community members (living either in Morwell or socioeconomically and demographically comparable areas of Sale and at least 18 years of age during the mine fire) were identified from the Victorian Electoral Commission roll. The present study took place among Cohort members in the third round of the Hazelwood Health Study's Respiratory Stream [20] and the long‐term respiratory follow‐up [21], who agreed to provide a DBS sample and answer questions about their COVID‐19 history during clinical visits between June and October 2023.

### 2.2 COVID‐19 Indicators

We used two measures of determining prior SARS‐CoV‐2 infection based on data collected during the 2023 clinical visit: a validated self‐report questionnaire and a serology test performed on a dried blood spot (DBS) sample.

The self‐report COVID‐19 questionnaire was validated by the Avon Longitudinal Study of Parents and Children [18]. It asked, “Do you think that you currently have or have had COVID‐19?”, and had four potential responses: (1) Yes, confirmed by a positive test; (2) Yes, suspected by a doctor but not tested; (3) Yes, my own suspicions; (4) “No.” We also asked participants about the year of their infection(s), allowing for multiples. Participants who answered “Yes” to any of the questions 1–3 were classified as having self‐reported COVID‐19. “Yes” respondents were subcategorized into the year of their most recent reported infection.

Serological samples were collected through DBS. Although venepuncture is the most common means of collecting blood samples for serology, it requires specialist training, cold chain storage, and timely processing of samples [22, 23, 24]. In contrast, DBS uses capillary blood drawn from a finger or heel prick, which is then applied to a piece of specialized filter paper. This technique requires minimal training and, after drying, antibodies in the samples are stable for months and can be stored and shipped in a plastic envelope at room temperature [22, 23, 24]. A growing body of research has demonstrated the validity of DBS against blood serum for detecting SARS‐CoV‐2 antibodies, with results comparable to venepuncture sampling [17, 24, 25, 26].

DBS samples were drawn with a finger prick. Up to five blood spots were placed onto a Guthrie Card, which was then dried, sealed, and stored at room temperature for up to 2 weeks before being transported to the Murdoch Children's Research Institute in Melbourne for analysis. The selected serology test was an Enzyme Linked Immunosorbent Assay (ELISA), which quantified IgG antibodies to SARS‐CoV‐2 nucleocapsid (N)‐protein in the blood, adapted from a previously published Spike (S) protein–specific IgG assay described below [17]. Estimates for post‐infection detectability of N‐protein antibodies in the long term vary considerably, including: 70% at 548 days post‐infection [27], 68% at 293 days [28], and 94% at 150 days [29], and 27% at 12 months [30]. Detectable antibody levels also vary depending on the assay used [31, 32, 33], as well as the severity of symptoms [30, 34, 35] and demographic characteristics [28, 36]. Compared to S‐protein antibodies, N‐protein serostatus decays much faster [28, 29], though in places like Australia that have only authorized component viral vaccines for COVID‐19, N‐protein antibodies can be used to distinguish infection from vaccination [27, 37]. This discrimination was essential as 97.4% of Australians aged 16 and over had received at least one dose of a COVID‐19 vaccination by January 2023 [38].

Seropositivity cut‐offs were established using pre‐pandemic samples [31], with seropositivity defined as the mean value plus two standard deviations. Samples with ELISA Units per milliliter (EU/mL) ≥ 30.94 were classified as seropositive, values < 21.52 EU/mL as seronegative, and in‐between as equivocal. For the main analyses, equivocal was treated as seronegative and seropositive in sensitivity analyses. Participants whose titers were too high to read were recoded to 1050 EU/mL.

### 2.3 Laboratory Procedures

#### 2.3.1 DBS Sample Processing

Briefly, a hole punch was used to produce 6 mm disks from the DBS (approximately 6 µL of serum). The disc was placed in a 1.5 mL Eppendorf tube, and 294 µL of elution buffer (PBS containing 0.1% Tween and 10% (w/v) skim milk) was added to the disc. Samples were eluted overnight with shaking at 350 rpm at room temperature. Eluted samples were centrifuged at 1000 × *g* for 5 min, and the supernatants were collected for serological testing.

### 2.4 SARS‐CoV‐2 N Protein ELISA

The SARS‐CoV‐2 N‐protein in‐house ELISA was adapted from a previously published Spike‐protein IgG assay [36]. Briefly, 96‐well high‐binding plates (Thermo Fisher Scientific) were coated with SARS‐CoV‐2 Nucleocapsid protein (Sino Biological) diluted in PBS at 2 µg/mL and then incubated at 4°C overnight. The following day, plates were washed with PBS containing 0.1% (v/v) Tween20 (PBS‐T) and blocked with PBS containing 0.1% Tween and 10% (w/v) skim milk (PBS‐TSM) for 1 h at room temperature. After incubation, the blocking solution was removed, and 50 µL of serial diluted DBS eluate was added to the plates for 2 h at room temperature. The plates were then washed three times with 200 µL per well of PBS‐T. Goat anti‐human IgG (1:10,000) horseradish peroxidase (HRP) conjugated secondary antibody (Southern Biotech) was prepared in PBS‐TSM, and 50 µL of this secondary antibody was added to each well for 1 h. Plates were washed with PBS‐T followed by distilled water and 50 µL of 3.3’, 5.5’‐tetramethylbenzidine (TMB, Sera Care) substrate solution was added for 9 min. The reaction was stopped by the addition of 50 µL of 1 M phosphoric acid, and optical densities were measured using a microplate reader (Bio‐Tek) at 450 nm (630 nm reference filter).

### 2.5 Statistical Analysis

We initially characterized the sample using descriptive statistics. Then, we compared the annual prevalence of self‐reported COVID‐19 in our sample to the annual incidence of cases in the local government areas of Latrobe City and Wellington (from which our sample was drawn), using data from the Victorian Department of Health [39] and Australian Bureau of Statistics [40].

To assess agreement between serostatus and self‐reported COVID‐19, we applied several statistical approaches. Here, we treated serostatus as the reference standard. First, we calculated Cohen's Kappa (κ), a measure of inter‐rater reliability that accounts for the likelihood of agreement by chance, to determine agreement between serostatus and self‐reported COVID‐19 in any year. Second, we calculated the positive and negative predictive values (PPV and NPV) of self‐reported COVID‐19, both overall and then by year of most recent self‐reported COVID‐19. Third, we conducted logistic regressions of the year of most recent self‐reported COVID‐19 on serostatus, both crude and adjusted for age at clinical visit (natural spline with 3 degrees of freedom to account for non‐linear effects), sex, and education level (secondary to year 10, secondary to year 11–12, certificate/trade/university qualification). The fourth approach was to conduct crude and adjusted linear regressions of N‐protein antibody concentrations, which were log‐transformed to present results as proportional change [41]. Finally, we performed sensitivity analyses that combined seropositive and equivocal antibody concentrations. Analyses using the year of the most recent self‐reported COVID‐19 combined 2020 and 2021 due to small numbers. Significance was set at ⍺ = 0.05 with 2‐sided tests. Analyses were conducted in R version 4.5.2 [42].

### 2.6 Ethics

This study was approved by the Monash University Human Research Ethics Committee as part of the Hazelwood Health Study: Respiratory Stream Round 3 (Project ID: 36471) and the Alfred Hospital Ethics Committee (project number 90/21). All participants provided written informed consent to participate in this study.

## 3 Results

### 3.1 Descriptives

Of the 821 members of the Hazelwood Health Study Adult Cohort invited to participate, 318 (39%) attended the clinic, and 312 (38%) provided DBS samples for serology. One had unreadable serology data and was therefore excluded from the analysis. Characteristics of the 311 participants included in the analysis are listed in Table 1.

**Table 1:** Participant characteristics.

Characteristic	Study sample (*n* = 311; *n* (%) or median [IQR])
Female	176 (57%)
Age	63 years [IQR: 52, 71]
N‐protein antibodies to SARS‐CoV‐2	9 EU/mL [IQR: 5, 43]
Seropositive for SARS‐CoV‐2 antibodies	98 (32%)
Equivocal	14 (4.5%)
Any self‐reported COVID‐19	203 (65%)
Confirmed by test	201 (65%)
Own suspicions	2 (0.6%)
Most recent year of self‐reported COVID‐19*	
2020	7 (2.3%)
2021	21 (6.8%)
2022	134 (43%)
2023	40 (13%)
Don't know	1 ( < 0.1%)
Never had COVID‐19	108 (35%)

* Total number of participants reporting COVID‐19 in each year (as opposed to most recent only) were as follow: 2020 – 7, 2021 − 33, 2022 − 151, 2023 − 40; self‐report and serology data were collected during a 2023 clinical visit; “most recent self‐reported COVID‐19” refers to retrospective answers of when infection occurred.

Two‐thirds (203, 65%) of respondents self‐reported a history of COVID‐19, with all but two reporting that the diagnosis was based on a positive test result. Conversely, one‐third (98, 32%) of participants were seropositive for SARS‐CoV‐2 N‐protein antibodies, indicating past infection. By year, most self‐reported infections were in 2022, corresponding to the biggest surge in infections observed in Victorian Department of Health data [39] and seropositive blood donors [43].

In Figure S1 (Appendix), we compare the study sample annual prevalence to population‐level annual incidence in Latrobe City and Wellington local government areas.^1^ Notably, while both have an outsized peak in 2022, the relative magnitude compared to other years was largest in the Latrobe City and Wellington population data. For instance, the 2022 annual incidence in Latrobe City and Wellington was 37.3%, 17.8 times greater than the next highest annual incidence year, which was 2.1% in 2023; in contrast, the equivalent figures for annual prevalence in the study sample was 48.6% in 2022 but only 3.7 times greater than 2023, where it was 12.9%.

### 3.2 Agreement Between Self‐Reported COVID‐19 and Serostatus

Across all years, the observed agreement between self‐reported COVID‐19 and seropositivity was 57%, compared to the expected agreement of 44% based on the crosstabs presented in Table 2. This can be summarized as “fair” agreement (κ = 0.23 [95% CI 0.15−0.31]) [44].

**Table 2:** 2 × 2 Table of COVID‐19 cases based on self‐report and serostatus.

	Seropositive	Seronegative	Totals	Predictive Agreement
Self‐report +	84	119	203	41%
Self‐report −	14	94	108	87%
Totals	98	213	311	

*Note:* Self‐report and serology data were collected during a 2023 clinical visit.

Using serostatus as a reference standard^2^, self‐reported COVID‐19 had a PPV of 41% and an NPV of 87% for SARS‐CoV‐2 seropositivity. These results are presented alongside the 2 × 2 contingency table in Table 2. The sensitivity analysis, which grouped equivocal with seropositive cases, did not meaningfully affect this level of agreement (κ = 0.24 [0.15−0.32]; see Table S1).

### 3.3 Agreement Between Self‐Reported COVID‐19 by Year and Serostatus

Table 3 presents the number of self‐reported COVID‐19 cases in each year by serostatus, along with the PPV. If a participant reported multiple infections, they were counted in the year of their most recent infection. NPV was not calculated for this analysis as ‘most recent self‐reported COVID‐19’ lacked true negatives (e.g., a participant whose most recent self‐reported COVID‐19 was in 2023 could also report COVID‐19 in a previous year). PPV was low for 2020‐21 (36%) and 2022 (33%), but higher (75%) for those whose most recent case was in 2023, which given the timeframe of data collection, would have been within 9 months of DBS sampling.

**Table 3:** Number of self‐reported COVID‐19 cases in each year by serostatus, and positive predictive value of the questionnaire using serostatus as a reference standard.

Most recent self‐reported COVID‐19	Seropositive	Seronegative	Positive predictive value
2020–21	10	18	36%
2022	44	90	33%
2023	30	10	75%

*Note:* Self‐report and serology data were collected during a 2023 clinical visit; “most recent self‐reported COVID‐19” refers to the respective answers of when infection occurred.

**Figure 1:**
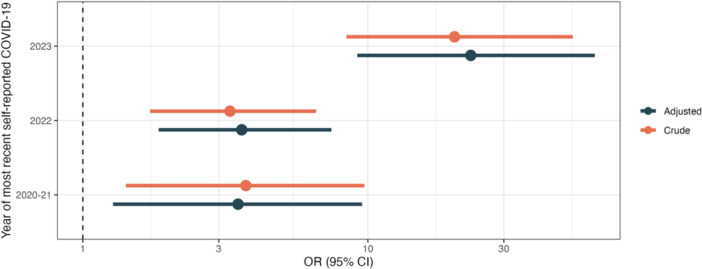
Crude and adjusted (age, sex, educational attainment) odds of seropositive dried blood spot test by year of most recent self‐reported COVID‐19; self‐report and serology data were collected during a 2023 clinical visit; “most recent self‐reported COVID‐19” refers to retrospective answers of when infection occurred.

In logistic regression analysis, those with the most recent SARS‐CoV‐2 infection in 2023 had 23 times greater odds of being seropositive (95% CI: 9.18–62.5) compared to those who self‐reported no history of COVID‐19, after adjusting for potential confounders; results were very similar to the crude associations, indicating limited confounding due to factors included in the model. Those whose most recent self‐reported infection occurred earlier were also positively associated with seropositivity, though the odds were lower. These results are illustrated in Figure 1 and model results in Table S2. Sensitivity analyses that grouped equivocal with seropositive cases were not meaningfully different (Figure S2 and Table S3).

### 3.4 Most Recent Self‐Reported COVID‐19 as Predictor of N‐Protein Antibody Concentration

**Figure 2:**
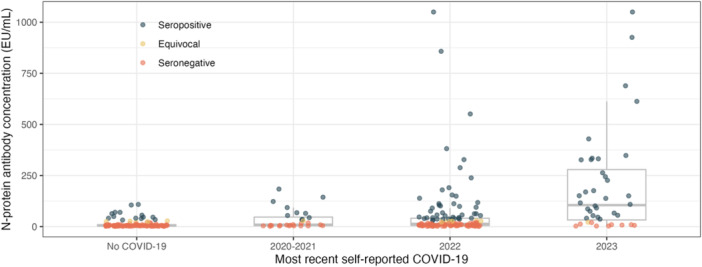
Distributions of antibody concentrations (EU/mL) and status by year of most recent self‐reported COVID‐19; self‐report and serology data were collected during a 2023 clinical visit; “most recent self‐reported COVID‐19” refers to retrospective answers of when infection occurred.

Distributions of N‐protein concentrations by year of most recent self‐reported COVID‐19 are illustrated in Figure 2. This shows much wider distributions and higher values in the 2023 group, and clustering around low values in the seronegative category.

Results of linear regressions are reported in Figure S3 and Table S4. Compared to participants with no self‐reported history of COVID‐19, those who reported their most‐recent case in 2020–2021 had 108% (95% CI: 18%–268%) greater N‐protein concentrations, those reporting their most recent case in 2022 had 151% (77%–255%) greater N‐protein concentrations, and those reporting their most recent case in 2023 had 1078% (616%–1836%) higher N‐protein concentrations, after adjustment for confounders.

## 4 Discussion

We found relatively weak agreement between self‐reported COVID‐19 and seropositivity. However, there were several interesting findings that could inform others looking for practical and affordable means to accurately determine whether study participants have a history of COVID‐19. First, those with more recent cases of self‐reported COVID‐19 had greater odds of being seropositive. This aligned with what is known about serum antibodies waning over the months following SARS‐CoV‐2 infection [11, 12, 13, 14]. Second, the questionnaire's NPV was high (87%), indicating those who reported no history of COVID‐19 were unlikely to have SARS‐CoV‐2 antibodies.

There are two instances in which we would expect a high degree of concordance if self‐reported COVID‐19 was reasonably accurate: among those with recent infections (when serology is most reliable) and those reporting no history of COVID‐19 (who should not be seropositive). We observed both in this study. The main source of discordance between COVID‐19 measures was among those with older infections, which was probably due to waning antibodies. By extrapolation, it may be justifiable to treat self‐reported COVID‐19 as accurate even for older infections since it performed well where we expected (high PPV with recent infections and high NPV overall) and poorly only where serology was less reliable (older infections). On the other hand, since seropositivity is related to disease severity [14], there will remain a gap in asymptomatic cases, which could lead to an overestimate of the accuracy of self‐reported COVID‐19.

Another challenge was relying on participants to accurately recall when they had COVID‐19. We added a question on the timing of illness to a previous questionnaire [21], but had not evaluated its validity. Our respondents reported a surge in infections in 2022, which was consistent with the recognized surge in cases regionally, likely due to the end of the lockdown era in Victoria, combined with the emergence of the highly infectious Omicron variant [45]. However, the relative magnitude of this surge compared to other years was considerably larger in the broader population of Latrobe and Wellington local government areas, suggesting some imprecision around *when* self‐reported infections occurred.

Our findings reflect the messy reality of capturing accurate health data, particularly in the context of COVID‐19. But researchers must do their best with resource constraints and available tools to still find meaning to guide policy and practice. One silver lining of this study was the performance of self‐reported COVID‐19 measures. While self‐reported COVID‐19 questions are not perfect (particularly for asymptomatic cases) and there was likely some inaccuracy in participants recalling when an infection occurred, they may nevertheless be a cost‐effective way to identify historical cases. This is valuable considering antibodies wane a few months after infection, particularly the SARS‐CoV‐2 N‐protein antibodies, and may render serology inaccurate within a few months of exposure. This study contributes to the understanding of the usefulness of both self‐report and serology sampling for COVID‐19 disease detection.

### 4.1 Strengths and Limitations

This study is novel in its evaluation of a validated COVID‐19 self‐report questionnaire in an Australian context. Australia is a high‐income country that had state‐subsidized PCR testing, thorough contact tracing, and relatively low case numbers prior to its vaccination program.

A limitation of this study was that neither of the two indicators of COVID‐19 that we compared were a true “gold standard”. It is also challenging to assess the impact of recall bias on questionnaire results over time, given that serology also becomes less reliable over time. Lastly, widespread testing in Australia in the early part of the pandemic may have provided participants with more objective data about their history of COVID‐19 than was available in countries that did not offer widespread testing, thus limiting this study's generalizability. While < 1% of our sample self‐reported COVID‐19 based on suspicions alone, the ALSPAC study (based in the UK, where infection rates were high before widespread testing or vaccination were available), found that a much larger proportion (12.8%) reported the diagnosis based on suspicion alone [18]. Furthermore, these findings may not be generalizable to younger age groups, in whom there tends to be a higher rate of asymptomatic or mild disease, or places where whole‐virus vaccines are commonly used, as the antibodies they produce are not usually serologically distinguishable from SARS‐CoV‐2 infection [37].

## 5 Conclusion

Our findings suggest that a validated COVID‐19 self‐report questionnaire is useful for identifying people who have previously had COVID‐19 and may be reasonably accurate in identifying when an infection occurred.

## Author Contributions

**Nicola Sheppard:** formal analysis (supporting), writing – original draft (lead). **Matthew T.C. Carroll:** conceptualisation (equal), funding acquisition (equal), methodology (equal), supervision (supporting), writing – review and editing (equal). **Brigitte M. Borg:** investigation (lead), resources (equal), supervision (supporting), writing – review and editing (equal). **Zheng Quan Toh:** data curation (equal), formal analysis (lead), investigation (supporting), methodology (equal), resources (equal), writing – review and editing (equal). **Paul V. Licciardi:** data curation (equal), formal analysis (supporting), investigation (supporting), methodology (equal), resources (equal), writing – review and editing (equal). **Catherine L. Smith:** formal analysis (supporting), validation (equal), writing – review and editing (equal). **Jillian F. Ikin:** funding acquisition (supporting), validation (equal), writing – review and editing (equal). **Michael J. Abramson:** conceptualisation (equal), funding acquisition (equal), methodology (equal), resources (equal), supervision (supporting), writing – review and editing (equal). **Karen Walker‐Bone:** funding acquisition (equal), resources (equal), writing – review and editing (equal). **Tyler J. Lane:** conceptualisation (equal), formal analysis (lead), methodology (equal), supervision (lead), visualisation (lead), writing – original draft (supporting), writing – review and editing (equal). All authors have read and approved the final version of the manuscript. Corresponding author Dr. Tyler Lane had full access to all of the data in this study and takes complete responsibility for the integrity of the data and the accuracy of the data analysis.

## Ethics Statement

This study was approved by the Monash University Human Research Ethics Committee as part of the Hazelwood Health Study: Respiratory Stream Round 3 (Project ID: 36471) and the Alfred Hospital Ethics Committee (project number 90/21). All participants provided written informed consent to participate in this study.

## Consent

All participants provided written informed consent to participate in this study.

## Conflicts of Interest

M.J.A. holds investigator‐initiated grants from Pfizer, Boehringer‐Ingelheim, Sanofi and GlaxoSmithKline for unrelated research. He has undertaken an unrelated consultancy for Sanofi and received a speaker's fee from GSK. The other authors declare no conflicts of interest.

## Transparency Statement

The lead author Tyler J. Lane affirms that this manuscript is an honest, accurate, and transparent account of the study being reported; that no important aspects of the study have been omitted; and that any discrepancies from the study as planned (and, if relevant, registered) have been explained.

## Supporting information


Supporting File:

## Data Availability

The data that support the findings of this study are available from the corresponding author upon reasonable request.

## Endnotes

1 While COVID‐19 annual incidence (number of cases in a population, which can include multiple infections per individual) and prevalence (persons within a population/sample who have had an infection, where each individual counts only once even if they have multiple infections in the same year) differ, they can be considered roughly comparable, especially as COVID‐19 imparts some short‐lived immunity to reduce the number of infections within a year.

2 The description of serostatus as “reference standard” is one of convenience to describe how the two measures to derive positive and negative predictive values.
